# Levels and determinants of continuum of care for maternal and newborn health in Cambodia-evidence from a population-based survey

**DOI:** 10.1186/s12884-015-0497-0

**Published:** 2015-03-19

**Authors:** Wenjuan Wang, Rathavuth Hong

**Affiliations:** International Health and Development Division, ICF International, Inc., 530 Gaither Road, Rockville, MD USA

**Keywords:** Cambodia, Maternal and newborn health, Continuum of care, Determinants

## Abstract

**Background:**

Continuum of care throughout pregnancy, birth, and after delivery has become a key program strategy for improving the health of mothers and newborns. Successful program implementation to improve the continuum of care relies on a better understanding of where the gaps are in seeking care along the pathway and what factors contribute to the gaps.

**Methods:**

Using data from the 2010 Cambodia Demographic and Health Survey, we examine the levels of service use along the continuum of care. Three sequential regression models are fitted to identify factor(s) that affect women from getting skilled birth attendance (SBA) after receiving antenatal care (ANC), and from getting postnatal care (PNC) after having both ANC and SBA.

**Results:**

Three of every five Cambodian women received all three types of maternal care—antenatal care, skilled birth attendance at birth and postnatal care -for their most recent birth, however with substantial regional variation ranging from 14% to 96%. The results highlight that mother’s age, educational attainment, urban residence, household wealth, lower birth order are associated with women’s use of antenatal care and their continuation to using skilled birth attendant. Health insurance coverage also increases use of antenatal care but not skilled birth attendant. Having four antenatal care visits and receiving better quality of antenatal care affected women’s subsequent use of skilled birth attendant. The odds of having skilled birth attendant increases by 30 to 50% for women who received blood pressure measurement, urine sample taken, and blood sample taken as part of antenatal services. Household wealth status, urine sample taken, and delivery at a health facility were the only three factors significantly associated with the continuation from having skilled birth attendant to receiving postnatal care.

**Conclusions:**

Cambodia has made remarkable progress in extending the reach of maternal health care in most areas of the country. Future program efforts should focus on the Northeast part of the country where the lowest level of service use was found. Poor women suffered from lower access to continued care and extending the health insurance coverage might be one way to help them out. Quality of antenatal care is connected to women’s use of skilled birth attendant and postnatal care and should be given more focus.

## Background

Cambodia has achieved remarkable progress in improving child survival in the past decade. Data from the 2010 Cambodia Demographic and Health Surveys (CDHS) show that under-five mortality has declined dramatically, from 124 deaths per 1,000 live births in 2000 to 54 per 1,000 live births in 2010 [1,2]. Infant mortality also declined from 95 to 45 deaths per 1,000 live births during this period. However, neonatal mortality in Cambodia has not changed much, at 28 deaths per 1,000 live births in 2005 and 27 per 1,000 live births in 2010. Neonatal mortality contributed to half of the total under-five mortality in 2010 compared with about 30% in 2000 and 2005. The country still has one of the highest levels of maternal mortality in the region- at 206 deaths per 100,000 live births.

High rates of maternal and neonatal mortality are associated with inadequate and poor-quality maternal health care, including antenatal, delivery, and postnatal care [3-5]. Antenatal care is considered as a key health maternal service in improving a wide range of health outcomes for women and children [3,6,7]. Skilled birth attendance referring to delivery care provided by an accredited health professional (a midwife, doctor or nurse) has been advocated as a key factor in reducing the risk of maternal death [8-10]. Postnatal care, especially within the first 48 hours after birth, is critical to the management of postpartum hemorrhage, a leading cause of maternal deaths in developing countries [4]. Antenatal care, skilled attendant at birth, and postnatal care within 2 days of birth are Countdown to 2015 indicators tracking progress towards Millenium Development Goals 4 and 5[11].

Given the importance of these three interventions, it is essential to provide all of them in a continuum of care to ensure the health of the mother and the newborn child. Although the term “continuum of care” for maternal, newborn health, and child health originally refers to continuity of care throughout the lifecycle—adolescence, pregnancy, childbirth, post-delivery period, and childhood [12], in this analysis we apply a narrowed scope of continuum of care, focusing on women during the period from pregnancy to childbirth and after delivery. The continuum of care has become one of the key program strategies for reducing maternal and newborn deaths and improving maternal and neonatal health and wellbeing [12]. Successful program implementation to improve the continuum of care relies on a better understanding of where the gaps are in seeking care along the pathway and what factors contribute to the gaps.

An extensive body of literature studied factors influencing the use of individual maternal health services. Research has found characteristics of women and their families, for example, maternal age at birth, parity, women’s education, employment status, household wealth are associated with the use of antenatal care and skilled birth attendant in various settings [13-16]. Some studies also identified relevant community-level factors including social norm and community media saturation [16]. Compared to antenatal care and skilled attendant at birth, research on postnatal care, however, is very limited in developing countries [17].

Instead of looking at maternal health services individually, we study from a perspective of continuum of care and aim to identify factors that affect women’s continuation in receiving care from pregnancy to childbirth and to after delivery. Research questions are: what proportions of women receive the three types of maternal health services that constitute the continuum of care—antenatal care, skilled birth attendance, and postnatal care? At what stage do women drop out of the course? What factors predict woman’s continuation of care along the pathway from pregnancy to the postnatal period?

## Methods

### Data

Data for this study come from the 2010 Cambodia DHS, a nationally representative survey. The CDHS adopted a two-stage sample design. The first stage involved randomly selecting clusters with probability proportional to size from a national master sample frame. At the second stage, a systematic sample of households was drawn from a listing of households in each of the sampled clusters. In the selected households, all women age 15–49 who had a live birth in the five years preceding the survey were interviewed about the care that they received during pregnancy, at and after delivery. We analyze 6,472 women and focus on the care they received for their most recent live birth.

### Measurements

The key measurements for this analysis are antenatal care, skilled birth attendance, and postnatal care. They are measured according to the WHO definitions. Antenatal care refers to pregnancy-related health care check-ups that a pregnant woman had either at a health facility or at home. Skilled birth attendance is defined as delivery assistance provided by a doctor, nurse, or midwife. Postnatal care usually comprises checkups by a health professional or others within 41 days or six weeks of childbirth. This analysis focuses on postnatal care for mothers within 48 hours after birth for two reasons: 1) first 48 hours after birth is critical to the management of postpartum hemorrhage, a leading cause of maternal deaths in developing countries; 2) postnatal care within 48 hours after birth is a Millennium Development Goal Countdown to 2015 indicator. Thus, references to postnatal care in the analysis should be interpreted as postnatal care within 48 hours after delivery. Data on postnatal care were collected for both women who delivered at a health facility and those who did not.

### Statistical analysis

We first describe the levels of service use along the continuum of care. We then fit three sequential regression models to identify factors predicting the continuation of care from ANC to SBA and then from SBA to PNC. As mentioned earlier, we focus on a narrowed scope of continuum of care, focusing on women during the period from pregnancy to childbirth and after delivery. To identify factors associated with use of antenatal care at the stage of pregnancy, we fit Model I among all studied women with receiving antenatal care as the outcome. It is coded 1 if a woman received any antenatal care and 0 otherwise. Among women who received antenatal care, some went on to receive skilled birth attendance; some did not. We fit Model II among women who received antenatal care to determine the factors associated with the continuity from having antenatal care to having skilled birth attendance. The outcome for Model II is 1 for receiving antenatal care and skilled birth attendance, and 0 for receiving antenatal care but not skilled birth attendance. After delivery, some women received postnatal care and some did not. Thus we fit Model III among women who received antenatal care and skilled birth attendance to identify factors associated with completion of the continuum of care. The two categories of the outcome for Model III are 1 for receiving antenatal care, skilled birth attendance, and postnatal care, and 0 for receiving antenatal care and skilled birth attendance but not postnatal care.

For all three models, random-effects logit regressions are fitted to account for the clustering effect of the data. The 2010 CDHS data are in a hierarchical structure with individuals nested within clusters and clusters nested within provinces. Women living in the same cluster or province may share similar characteristics and behaviors; hence the estimates from regular regression analysis assuming all individual are independent will not be efficient. The random-effects model accounts for the fact that people who live in the same area share similar characteristics [18]. The random-effects model also enables partitioning of the total variation in the outcome into within-group and between-group components, which allows distinguishing the relative contributions of individual-level and group-level variables. In this analysis we are interested in two levels—the individual level and the province level. Despite the unavailability of provincial-level predictors in this analysis, random-effects models provide information on the proportion of total variation that is explained by provincial factors (unobserved).

Predictors in the models include women’s socio-demographic characteristics, urban–rural residence, household wealth quintile, health insurance coverage, and exposure to mass media. These factors have been shown in the literature to be correlated with the use of maternal health services [19-23]. It is also expected that the content of antenatal care, an indication of the quality of care is associated with use of delivery care and postnatal care and the place of delivery is associated with postnatal care [24]. Therefore, variables related to services received during antenatal care are included in Model II and Model III, and place of delivery is also included in Model III.

We first examine unadjusted associations between use of maternal health services and explanatory variables based on Chi-square test. The significant variables (p < 0.05) are then entered into multiple logistic regressions to assess the associations after adjusting for other variables. Once variables are entered in the model, they remain there unconditionally. Sample weights are accounted for in both descriptive and regression analyses.

### Ethics

The Institutional Review Board of ICF International, Inc. reviewed and approved the MEASURE Demographic and Health Surveys Project Phase VI, and the 2010 Cambodia Demographic and Health Survey is categorized under that approval. The Institutional Review Board of ICF International complied with the United States Department of Health and Human Services regulations for the protection of human research subjects (45 CFR 46).

## Results

Table 1 shows background characteristics of the women analyzed. Most women included in this study had their most recent birth at age 20–34, already had one or more children, and had some education. The majority lived in rural areas and 80% reported having regular access to various types of media. Access to health insurance was low, at 14%.

**Table 1:** **Background characteristics of women who had a live birth in the five years preceding the survey, Cambodia 2010**

	**Number of women**	**Percentage**
Mother’s age at birth		
<20	555	8.6
20-34	4,917	76.0
35+	999	15.4
Birth order		
1	1,980	30.6
2	1,786	27.6
3	1,146	17.7
4 or more	1,561	24.1
Mother’s education		
None	1,133	17.5
Primary	3,635	56.2
Secondary or higher	1,703	26.3
Residence		
Urban	1,050	16.2
Rural	5,421	83.8
Wealth quintile		
Lowest	1,585	24.5
Second	1,380	21.3
Middle	1,229	19.0
Fourth	1,155	17.8
Highest	1,123	17.4
Exposure to mass media^1^		
Yes	4,020	62.1
No	2,452	37.9
Health insurance coverage		
Covered by a health insurance	875	13.5
Not covered	5,596	86.5
Total	6,472	100.0

^1^Refers to read a newspaper or watch TV or listen to radio at least once a week.

### Overall use of maternal health services in Cambodia

Cambodia has achieved a high rate of antenatal care coverage. Over 90% of women who had a live birth in the five years preceding the survey had at least one antenatal care visit during the pregnancy, and nearly 60% had the WHO-recommended four or more visits. Compared with other countries in the region, Cambodia has one of the highest antenatal care coverage rates.

In Cambodia 74% of the women were attended by a skilled health provider (doctor, nurse, or midwife) at delivery for their most recent birth in the five years preceding the survey. Among these women, 57% reported giving the birth at a health facility—47% at public health facility and 10% at private health facility. Almost all women who delivered at a health facility were attended by a doctor, nurse or midwife. In contrast, among women who gave birth at home, 39% were attended by a skilled health provider (mostly by a midwife) and the rest were mainly attended by a traditional birth attendant.

For the postnatal care, 71% of women, regardless where they delivered, received a health checkup within two days of delivery, of which 87% had the first checkup within the first 24 hours. It is expected to observe most women (90%) who delivered at health facilities had postnatal. However, even those women who delivered at home, about one-quarter of them also had a doctor or midwife checked on them within 48 hours.

### Continuum of care

**Figure 1:**
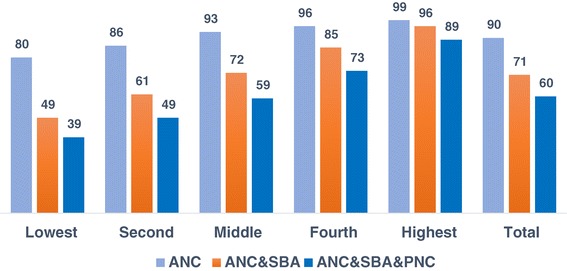
**Continuum of maternal health care by wealth quintile – percentage of women who received care by household wealth quintile.**

Figure 1 shows that while 90% of women received antenatal care, 71% continued for skilled birth attendance, meaning 19% dropped on the pathway to receive. After delivery, another 11% did not go on to receive postnatal care. Overall, 60% of women had the full range of services for the continuum of care. The use of services substantially varies by wealth status-89% of women from the richest households, compared with only 39% of those from the poorest households received the full range of services.

Table 2 shows the percentages of women who received the various possible combinations of maternal services. Two groups deserve more attention − 6% of women who did not receive any of the three services and 12% had only antenatal care but not the other two. As one might expect that few women received skilled birth attendance or postnatal care or both without first having received antenatal care.

**Table 2:** **Percent distribution of women by different types of maternal health services received for the most recent birth, Cambodia 2010**

**ANC**	**SBA**	**PNC**	**%**
**-**	**-**	**-**	5.5
+	-	-	11.9
+	+	-	10.9
+	+	+	59.8
+	-	+	7.2
-	+	-	1.2
-	-	+	1.5
-	+	+	2.1
Total			100.0
Total number of women			6,472

**+** Received the service.

**-** Did not receive the service.

ANC = antenatal care, SBA = skilled birth attendance, PNC = postnatal care.

**Figure 2:**
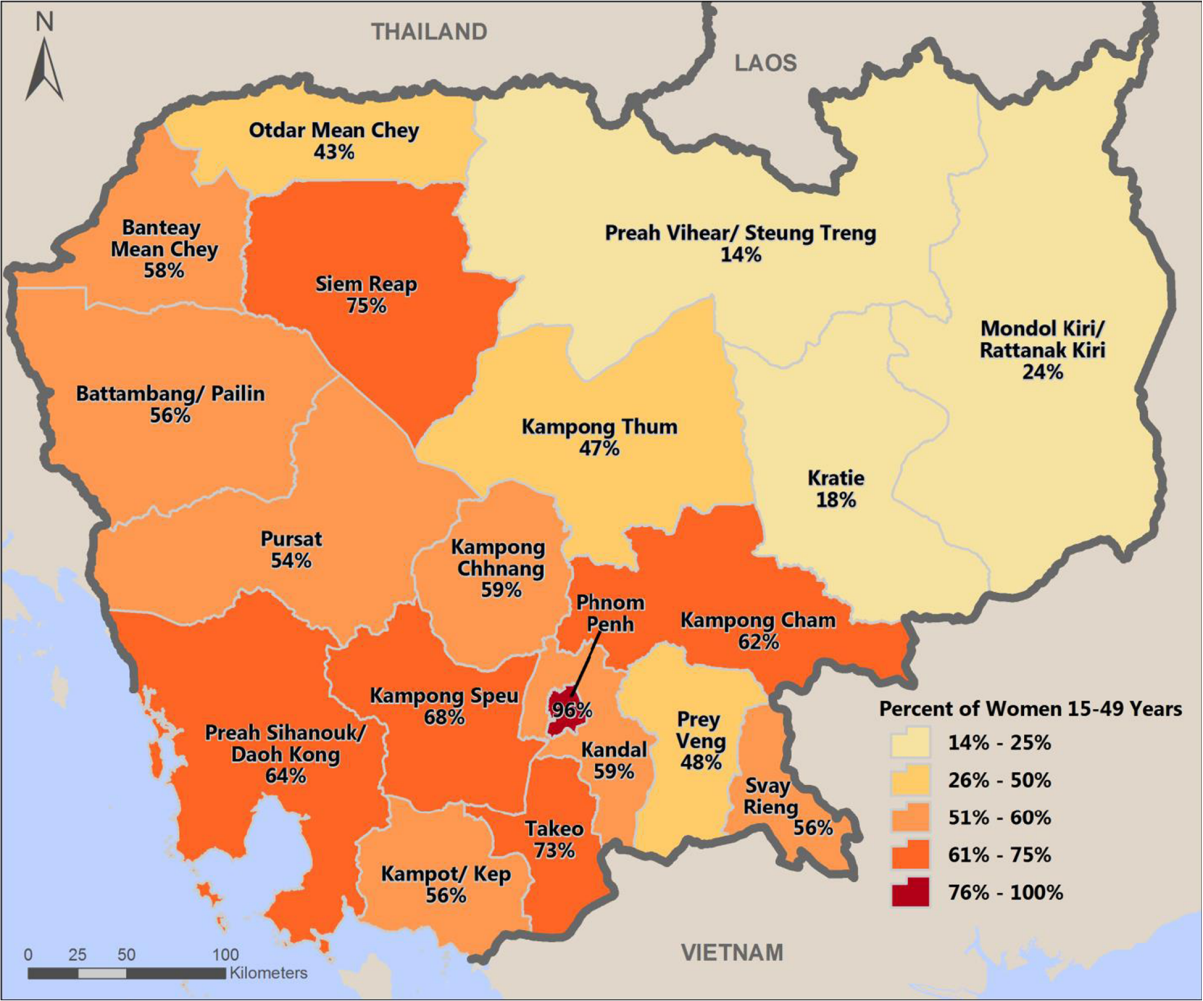
**Completion of the continuum of care by provinces.**

Cambodia’s regions varied substantially in the completeness of care. Figure 2 shows the percentage of women in each province who received all three services. In the capital city, Phnom Penh, 96% of women received antenatal care, skilled birth attendance, and postnatal care. Women in several other provinces around Phnom Penh—Takeo, Kampong Speu, Preah Sihanouk/Daoh Kong, and Kampong Cham—also reported receiving continuity of care, ranging from 62 to 73%. Three provinces in the northeast, however—Preah Vihear/Steung Treng, Mondol Kiri/Ratttanak Kiri, and Kratie—had the lowest percentage of women completing the continuum of care. These three provinces also reported the lowest level of use for each individual service. For example, in Preah Vihear/Steung Treng only 32% of women reported skilled birth attendance and 23% reported postnatal care, far below the national averages.

### Regression analysis results

As described in the methods section, three random-effects logit regression models are fitted to identify facilitators (or inhibitors) for women to receive maternal health services along the continuum of care. The results are shown in Table 3. In each model *rho* is the intra-class correlation coefficient, which measures the proportion of variation in the use of maternal services that is between provinces.

**Table 3:** **Results of the multivariate regression models**

	**Model I**		**Model II**		**Model III**	
	**ANC**		**ANC & SBA**		**ANC & SBA & PNC**	
	**Odds ratio**	**95% CI**	**Odds ratio**	**95% CI**	**Odds ratio**	**95% CI**
Mother’s age at birth (ref. = age <20)						
20-34	1.7***	1.2 - 2.4	1.3*	1.0 - 1.8	1.0	0.7 - 1.4
35+	1.2	0.8 - 1.9	1.5**	1.0 - 2.2	0.9	0.6 - 1.5
Birth order (ref. = 1)						
2	0.6***	0.4 - 0.8	0.8**	0.6 - 1.0	1.2	1.0 - 1.6
3	0.4***	0.3 - 0.6	0.7***	0.5 - 0.9	1.4	1.0 - 1.8
4 or more	0.3***	0.2 - 0.4	0.5***	0.4 - 0.7	1.1	0.8 - 1.6
Mother’s education (ref. = no education)						
Primary	2.0***	1.7 - 2.4	1.5***	1.3 - 1.9	1.1	0.9 - 1.5
secondary or higher	4.8***	3.3 - 7.0	2.8***	2.1 - 3.6	1.2	0.9 - 1.7
Residence (ref. = urban)						
Rural	0.8*	0.6 - 1.0	0.4***	0.3 - 0.5	1.0	0.8 - 1.3
Wealth quintile (ref. = lowest)						
Second	1.5***	1.2 - 1.9	1.2**	1.0 - 1.5	1.4**	1.0 - 1.8
Middle	2.3***	1.8 - 3.0	1.7***	1.3 - 2.1	1.4**	1.0 - 1.9
Fourth	3.0***	2.2 - 4.2	2.9***	2.2 - 3.8	1.9***	1.4 - 2.7
Highest	6.5***	3.9 - 10.9	7.8***	5.1 - 12.1	2.7***	1.8 - 3.9
Exposure to FP messages (ref. = no)						
Yes	1.4***	1.2 - 1.7	1.0	0.8 - 1.2	0.9	0.7 - 1.1
Health insurance coverage (ref. = no)						
Yes	1.3***	1.1 - 1.7	1.2	1.0 - 1.4	1.1	0.8 - 1.4
Had 4 or more ANC visits (ref. = no)						
Yes			2.0***	1.7 - 2.4	1.2	1.0 - 1.4
Informed of signs of complications (ref. = no)						
Yes			1.0	0.9 - 1.3	1.2	1.0 - 1.5
Blood pressure measured (ref. = no)						
Yes			1.3**	1.1 - 1.7	1.1	0.8 - 1.5
Urine sample taken (ref. = no)						
Yes			1.5***	1.2 - 1.8	1.6***	1.3 - 1.9
Blood sample taken (ref. = no)						
Yes			1.4***	1.2 - 1.6	1.1	0.9 - 1.4
Provider of antenatal care^1^ (ref. = doctor)						
Nurse/midwife			1.0	0.6-1.5		
Traditional birth attendant or others			0.3**	0.1-0.8		
Delivery at a facility (ref. = No)						
Yes					6.4***	5.2 - 7.9
rho	0.12***		0.10***		0.35***	
Number of women	6,472		5,804		4,571	

^1^If more than one provider of ANC was mentioned, only the provider with the highest qualifications is considered. ***p < 0.01, **p < 0.05.

Model I analyzes factors associated with use of antenatal care. The results show that use of antenatal care is significantly associated with age 25–34, lower birth order, educational attainment, urban residence, wealth, health insurance coverage, and exposure to mass media.

Education and household wealth have relatively stronger effects than the other predictors. The odds of using antenatal care are almost five times higher for women with secondary or higher education than for women with no education. A woman covered by a health insurance has 30% greater odds of having antenatal care. Regular access to mass media (reading a newspaper, or watching television, or listening to radio at least once a week) increases the odds of antenatal care by 40%. In this model *rho* is 0.12, meaning that between-province variation accounts for 12% of the total variation in use of antenatal care. That is, most of the variation in antenatal use is attributable to differences in individual characteristics rather than residence in a particular province.

Model II analyzes the predictors of continuation of care from pregnancy to delivery among women who received antenatal care. Variables related to antenatal care services (number of visits, indicators of the quality of care, and provider of care) are added into this model in addition to those included in Model I. All of the variables remain significant except for exposure to mass media and insurance coverage. The results from model II indicate the importance of having four or more antenatal care visits and receiving higher quality of antenatal care for subsequent use of skilled birth attendants. Women with four or more antenatal visits have twice the odds of receiving skilled birth attendance compared with women with fewer than four visits. The odds of having skilled birth attendance increases by 30% to 50% for women of whom blood pressure was measured, urine sample was taken, and blood sample was taken as part of antenatal services. *Rho* in this model is 0.10, meaning that between-province variation accounts for just 10% of the total variation in continuation from antenatal care to skilled birth attendance.

Model III estimates the effects of predicators on the continuation of care from delivery to the post-delivery period among women who received both antenatal care and skilled birth attendance. All of the variables in Model II stay and place of delivery (delivery at a health facility or not) is added to Model III. Only three predictors are significant in this model—household wealth, urine sample taken during antenatal care, and place of delivery. Women from wealthier households are more likely to receive postnatal care than women from poorer households, although the effect of wealth on postnatal care does not appear to be as strong as its effect on antenatal care and skilled birth attendance. Delivering in a health facility is strongly associated with postnatal care—at over six times higher compared with not delivery in a health facility. Individual variables do not appear to explain much variation in use of postnatal care.

*Rho* in this model is 0.35, much larger than in the other two models, which indicates that differences between provinces account for more than one-third of the total variation in use of postnatal care among women who received both antenatal care and skilled birth attendance.

## Discussion

Continuum of care has become a key strategy of intervention programs for improving health and wellbeing of mothers and newborns. This strategy calls for a service delivery system connecting the three components of maternal care—antenatal, delivery, and postnatal services—with high-quality services at each of these levels. To facilitate designing and implementing such a system, it is necessary to understand where a country stands in the continuum of care, where women are lost along the pathway from one service to the next, and what should be the focus of efforts to improve the continuity of care.

Cambodia has been successful in extending coverage of important maternal health interventions. Three of every five Cambodian women were able to continue receiving key maternal care from the pregnancy to post delivery. This is a remarkable achievement for a country where 30% of the population lives below the poverty line. It reflects the program efforts of the government in collaboration with multiple international development partners to improve maternal care [25].

Nonetheless, the study found that after receiving antenatal care many women dropped out from the pathway of continued care and did not have a skilled birth attendant or postnatal care. More dropouts occurred between pregnancy and delivery than between delivery and the postnatal period. Receiving four ANC visits and quality of antenatal care were found to be significant predictor of subsequent use of skilled birth attendant. In a recent multi-country analysis, Adjiwanou and Legrand had similar findings that antenatal care has a positive effect on skilled birth attendance and the effect is mediated by the actual care women received during ANC visits [26]. When women receive high-quality antenatal care, they become better informed about pregnancy and more likely to recognize the importance of skilled delivery care. WHO recommended that women should receive focused antenatal care covering a range of essential services at certain times during pregnancy [27]. Delivering these service requires more than one ANC visit. Further programs in Cambodia should focus on increasing number of visits as well as the actual care provided to women.

Most of women who had skilled birth attendant at delivery continued to receive postnatal care. Although this finding could be partially due to women’s misreport of postnatal care as discussed later in the limitation section, it is expected that women are more likely to be checked within 48 hours of delivery when they were attended by a skilled health provider. The chance is even greater when women delivered at a health facility. These findings demonstrate that increasing the use of skilled birth attendant, and especially delivery at health facilities, could lead to more use of postnatal care and thus improve the continuity of care in Cambodia.

The study found substantial socioeconomic disparities in use of maternal health services in Cambodia. Women from the wealthiest households are over seven times more likely to report use of antenatal care and eight times more likely to report both antenatal care and skilled birth attendance compared with women from the poorest households. Although health insurance enrollment would be expected to improve access to health services in general, this study shows that health insurance coverage only affects women’s use of antenatal care but not use of both antenatal care and skilled birth attendants and use of all three services together. Health insurance in Cambodia is still at the early stage and has limited nationwide coverage. Health Equity Funds, as the main insurance scheme, have primarily focused on several provinces, where they have started to have an effect, based on small-scale evaluations [28].

Another interesting finding is that the sizable difference between women received skilled birth attendance and those who delivered at health facilities (74% versus 57%), which differs from the pattern usually seen in other countries, where the two groups are quite similar in size [29]. The fact that a substantial proportion of Cambodian women surveyed were attended by a skilled health provider even though they did not deliver at a health facility is in part because many health professionals, including doctors and midwives, who are employed in the public sector also practice in the private sector, to compensate for the low salaries offered by the public sector [30]. While this practice might improve health service coverage, it could cause problems for the public sector, which plays a dominant role in providing maternal health care services in Cambodia. Over 80% of women who received antenatal care and over 90% of women who delivered at health facilities reported receiving these services in the public sector. When health workers are busy with their own practice, they may not be available during work hours, or the quality of care in public facilities might be compromised [31]. Moreover, the quality of care that private practitioners provide is unknown. There is a concern that they may not adhere to treatment guidelines, due to lack of regulation [32]. The private sector is an important component of Cambodia’s health system, as a major source of general health care. Thus the government may work toward building a public-private partnership in providing maternal health care and also ensuring the quality of care.

Among women who received antenatal care and skilled birth attendance, very few factors were identified to be associated with continuation to postnatal care, while differences by province account for a larger proportion. The regional variation in use of maternal health services overall is large. By mapping the level of continuum of care in Cambodia’s provinces, we found geographic clustering of high use and low use of maternal services. High-use provinces are located around the capital city, where the use of all three services is almost universal. Its surrounding provinces—Takeo, Kampong Speu, and Kampong Cha—also demonstrate high levels of completion of continuum of care. These three provinces are more socioeconomically developed and have better access to a large number of health providers, including both public and private services. In contrast, three provinces in the northeast (Preah Vihear/Steung Treng, Kratie, and Mondol Kiri/Rattanak Kiri) have the lowest use of maternal services. These three provinces have relatively small population dispersed in a large mountainous area and have very limited health resources available. More program efforts should be directed to these regions.

The study is subject to several limitations. The analysis uses secondary data; therefore we could not include other factors such as maternal complications that could affect use of maternal care. The quality of data on postnatal care is subject to women’s misreport. Women could misreport care that was part of delivery care as postnatal care. Moreover, the way that the question is phrased “…*did anyone check on your health*” may not be clear enough for many women to understand [33]. Using 48 hours after delivery as the cutoff in defining postnatal care could potentially attenuate the effects of some covariates that increase women’s propensity to give birth at health facility. This is because when a women delivers at a health facility, she is more likely to be seen within 48 hours after delivery.

## Conclusions

A large proportion of Cambodian women complete the continuum of care from pregnancy to post delivery. Future program efforts should focus on the Northeast part of the country where the lowest level of service use was found. Poor women suffered from lower access to continued care. Continued efforts including increasing health insurance coverage should be invested to reduce inequalities in coverages. Quality of antenatal care is connected to women’s use of skilled birth attendant and postnatal care and should be given more focus.
